# Performance and Metabolic Responses of Nellore Cows Subjected to Different Supplementation Plans during Prepartum

**DOI:** 10.3390/ani14162283

**Published:** 2024-08-06

**Authors:** Douglas Teixeira Saraiva, Samira Silveira Moreira, Mateus Emanuel Pereira Santos, Eduarda Ramos Almeida, Luciana Navajas Rennó, Sebastião de Campos Valadares Filho, Mário Fonseca Paulino, Érica de Paula Aniceto, Johnnatan Castro Cabral Gonçalves, Jean Marcelo Albuquerque, Sidnei Antônio Lopes

**Affiliations:** 1 Departament of Animal Science, Universidade Federal de Viçosa, Viçosa 36570-900, MG, Brazil; mateuseps@hotmail.com (M.E.P.S.); lucianarenno@ufv.br (L.N.R.); scvfilho@ufv.br (S.d.C.V.F.); mpaulino@ufv.br (M.F.P.); ericadepaulaaniceto@gmail.com (É.d.P.A.); johnkbral@gmail.com (J.C.C.G.); jean.albuquerque@ufv.br (J.M.A.); sidnei.lopes@ufv.br (S.A.L.); 2 Department of Veterinary Medicine, Universidade Federal de Viçosa, Viçosa 36570-900, MG, Brazil; eduardaramos.vet@gmail.com

**Keywords:** nutrition, metabolism, reproduction

## Abstract

**Simple Summary:**

This study examined the effects of different prepartum supplementation plans for Nellore cows, testing a control group receiving only minerals and three groups receiving 2 g, 4 g, or 6 g of protein-energy supplement per kilogram of body weight daily. Cows receiving 4 g and 6 g supplements showed improved body weight, condition scores, and a shorter service period. Higher supplement levels also enhanced some metabolic health markers. We recommend 4 g of supplement per kilogram of body weight to improve overall metabolic health and performance.

**Abstract:**

This study assessed the effects of different prepartum supplementation plans on Nellore cows’ performance, metabolic responses, and early offspring development. Thirty-nine pregnant Nellore cows (224 ± 2.67 days of pregnancy, 5.3 ± 0.29 years of age, body weight 520 ± 15.2 kg, initial body condition score 6.0 ± 0.07) were assigned to one of four treatments: a control group receiving only mineral mixture ad libitum, and three groups receiving daily protein-energy supplements of 2, 4, or 6 g/kg BW for 60 days prepartum. Weights and body condition scores were evaluated at the start of the experiment, 7 days before calving, and at 45 and 90 days postpartum. Cows supplemented with 4 and 6 g/kg BW showed improved body weight and body condition scores prepartum and postpartum and had a shorter service period (*p* < 0.05). The highest blood urea nitrogen concentrations were observed in cows receiving 6 g/kg BW (*p* = 0.0124). There was a reduction in blood urea nitrogen at calving for the 6 g/kg BW group, while the control group showed an increase (*p* < 0.001). Non-esterified fatty acids concentrations were lower 21 days before calving for the 4 and 6 g/kg BW groups compared to the control (*p* < 0.05) and decreased postpartum for all treatments (*p* < 0.001). No significant differences were observed in calf birth weight or performance. Supplementing with 4 g/kg BW of protein-energy is recommended to enhance metabolic health and overall performance.

## 1. Introduction

The nutritional status of beef cows at calving is the main factor influencing the service period [1,2]. An inadequate supply of nutrients at the end of pregnancy is detrimental to reproduction, even when it is provided in sufficient quantity during lactation. There are studies that show that supplementing cows in the pre-calving period is more important than in the post-calving period [3,4,5].

In beef cattle production systems, the reproductive season is ideally timed when body weight, body condition score (BCS), and nutritional levels are at their optimal levels. However, beef cows spend most of their gestation period during the dry season, which is characterized by low availability and quality of forage. During this period, it is recommended that forage availability should be in the upper range of 4 to 6% of body weight (BW), with a crude protein (CP) content above 70 to 80 g/kg of dry matter (DM). Thus, supplementation during this period can correct nutritional deficiencies and improve cows’ body condition [6,7], as this is one of the most important factors in determining the extent of postpartum anestrus [8]. On the other hand, excess nutrients in the prepartum period have also been associated with adverse effects on reproductive efficiency [5,9,10] and an increased incidence of dystocia.

Changes in the nutritional status of beef cattle can be assessed through metabolites that link nutrition and physiology [11] and help to accurately indicate the effects of supplementation on animal metabolism. However, studies with Bos indicus under pasture conditions are scarce and the results have been inconsistent. In a study of grazing Nellore cows receiving protein-energy supplementation during the last 60 days of gestation, ref. [12] observed an improvement in the prepartum negative energy balance. However, no residual postpartum effects were detected. In contrast, ref. [13] reported that prepartum supplementation with a protein-rich concentrate led to increased body weight in cows and higher birth weight in calves.

The hypothesis is that higher levels of pre-calving supplementation reduce the negative energy balance and improve reproduction in Nellore cows. Therefore, the aim of this study was to evaluate different nutritional plans for beef cows in prepartum and their effects on performance, metabolism, and influence on the early development of calves.

## 2. Materials and Methods

### 2.1. Animals, Experimental Design and Treatments

All practices involving the use of animals were approved by the Ethics Committee on Animal Use at the Federal University of Viçosa (Protocol CEUAP-UFV 142/19).

**Figure 1:**
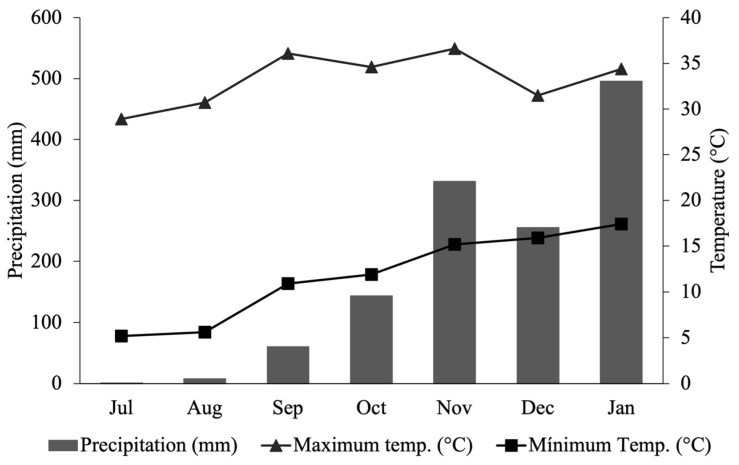
Precipitation and average temperature during the experimental period. Viçosa—MG. Source: INMET.

The experiment was conducted at the Teaching, Research, and Extension Unit in Cattle (UEPE-GC), at the Federal University of Viçosa, Minas Gerais, Brazil (20°45′ S 42°52′ W). The research was conducted from July 2019 to January 2020. The mean values of temperature were 22.5 °C and the total precipitation was 1295 mm (Figure 1).

We used 39 pluriparous, pregnant female Nellore cows with an average of 224 ± 2.67 days of pregnancy at the start of supplementation, 5.3 ± 0.29 years of age, a body weight (BW) of 520 ± 15.2 kg, and a mean initial body condition score (BCS) of 6.0 ± 0.07, as detailed in the following section. Body weight (BW) was measured individually using a mechanical scale (Valfran^®^, Votuporanga, Brazil) at both the beginning and end of the experiment.

The animals were randomly assigned to a completely randomized design and underwent 14 days of adaptation to the experimental area to be divided into lots of 4 or 5 animals each, and also to the diet.

The experimental area consisted of eight paddocks of approximately 7.5 hectares each, covered with *Urochloa decumbens* grass, with free access to water and feeders. Supplementation was 2 g/kg BW for all the animals, including those that would be allocated to the control group in the future. During the adaptation period, endo- and ectoparasites were also controlled with 3.5% doramectin (Treo ACE^®^, Zoetis, SP, Brazil).

The nutritional plans evaluated were control (cows that received only mineral mixture ad libitum) and groups of cows that received 2, 4, or 6 g/kg body weight of protein-energy supplement for 60 days before calving, in addition to mineral mixture ad libitum. The supplement was provided in groups. After calving, all groups received only mineral mixture ad libitum until 90 days after calving. The supplement was formulated to contain 300 g of crude protein per kg of dry matter (Table 1 and Table 2).

Supplements were provided daily at 11:00 a.m. To prevent paddock effects in the treatments the animals were alternated between paddocks every seven days so that each group remained for the same amount of time in each paddock. Supplements were supplied to the group of animals, with the amount calculated in relation to the group’s average weight.

### 2.2. Performance

Body Condition Score (BCS) is a tool used to assess the amount of body fat and muscle reserves in an animal through visual evaluation. This score typically ranges on a scale from 1 to 9, where 1 indicates a very thin animal and 9 indicates an obese animal [14]. To evaluate the performance of the cows, weights and BCS evaluations were performed at the beginning of the experiment (60 days prepartum) and 7 days before the expected calving date, and at 45 and 90 days after calving. The BCS was assessed by 3 experienced evaluators to NRC [14]. Body weight change prepartum was calculated as the difference in BW 7 days before calving compared to the initial weight, and weight change postpartum was calculated as the difference in BW at 45 and 90 days compared to calving. Shortly after birth, the calves received their first navel treatments, identification, and weighing at birth and at 90 days after birth to assess average daily gain (ADG).

The cows, 45 days after calving, underwent the mating season, during which they were synchronized and then performed fixed-time artificial insemination (FTAI). The same protocol was used for all animals, which consisted of the insertion of an intravaginal device containing 1 g of progesterone (P4) (PRIMER^®^, Agener Union animal health, São Paulo (SP), Brazil) on the first day of the protocol (D0), together with the administration of 2 mg of estradiol benzoate (RIC BE^®^, Agener União animal health, SP, Brazil), via the intramuscular administration. After 8 days (D8), the intravaginally P4 device was removed and 0.524 mg of Cloprostenol (ESTRON^®^, Agener Union Animal helth, SP, Brazil) and 300UI of equine chorionic gonadotropin (ECEGON^®^, Biogénesis Bagó animal health, Curitiba, Brazil) were administered via intramuscular injection. On day 9 (D9), we applied 1 mg of estradiol benzoate (RIC BE^®^, Agener Union animal health, SP, Brazil) and it was also administered via intramuscular injection. 

Artificial insemination (AI) was scheduled according to preovulatory follicle diameter (PFOD) for 48 or 56 h after P4 device removal. Animals with PFOD ≥ 14 mm at D10 were inseminated at the same time (48 h), while those with PFOD < 14 mm were evaluated again after 8 h when they were then inseminated (56 h), provided that follicular growth or ovulation was observed. Animals that presented PFOD less than 11.5 mm or more than 20 mm in any of the evaluations received 25 μg of Lecirelina, a GnRH analog (TEC-Relin^®^, Agener Union animal health, SP, Brazil). All cows were examined via transrectal ultrasound 30 days after the first fixed-time artificial insemination (FTAI). Those who were not pregnant underwent a second FTAI and were re-evaluated 30 days later. Cows with a viable embryo were classified as pregnant. Pregnancy at the first FTAI was calculated as the proportion of pregnant females 30 days after the first FTAI, divided by the total number of cows, and overall pregnancy was calculated as the proportion of pregnant females 60 days after the second FTAI, divided by the total number of cows. The service period was calculated as the number of days it took the animals to become pregnant.

### 2.3. Body Composition

Seven days before the expected calving date, body composition was measured using ultrasound to measure the loin eye area (LEA), in cm^2^, thickness of subcutaneous rib fat (TSR), in mm, and thickness of subcutaneous fat on the croup (TSC) in mm. The TSC images were taken between the ileum and ischium in a rectilinear position between the two tuberosities until the identification of the upper border of the Biceps femuris, while the TSR and LEA images were obtained in the intercostal region, between the 12th and 13th ribs, in the middle distal third of the loin eye area. The equipment used was the Aloka ultrasound (SSD 500V^®^, Aloka, Ltd., Tokyo, Japan) with an 18 cm linear probe. The images were analyzed with BioSoft Toolbox^®^ II for beef (Biotronics Inc., Ames, IA, USA).

### 2.4. Forage Sampling

Pasture samples were collected every 30 days by handpicking (simulated grazing) to assess forage quality.

To evaluate forage availability, forage was collected by cutting 5 cm from the ground at five random points in each paddock, using a metal square of dimensions (0.5 × 0.5 m) every 30 days. Subsequently, all samples were weighed, dried in an oven (55 °C), and then ground in a Willey mill (Thomas-Wiley Laboratory Mill Model 3: Arthur H. Thomas Co., Philadelphia, PA, USA) at 2 and 1 mm for further analysis.

Forage and supplement samples processed on a 1 mm sieve were analyzed following the description of standard analytical procedures of the National Institute of Science and Technology in Animal Science (INCT-CA) [15], for the content of DM (INCT-CA method G-003/1), crude protein (CP; INCT-CA method N-001/1), mineral matter (MM; INCT-CA method M-001/1), ether extract (EE; INCT-CA method G-004/1), neutral detergent fiber, using thermostable alpha amylase without the addition of sodium sulfite and corrected for ash and protein (apNDF; INCT-CA method F-002/1). For quantification of indigestible neutral detergent fiber (iNDF; INCT-CA method F-009/1), an incubation procedure was performed in situ nonwoven textile bags (100 g/m^2^) for 288 h of the samples processed at 2 mm. 

### 2.5. Milk Sampling

To estimate milk production the cows were milked 30 days postpartum using a milking machine. In order to drain the udders of the cows, the calves were separated from their mothers from 3:00 p.m. to 5:45 p.m. when they were reunited with their mothers so that they could suckle and then drain the milk from their udders. At 6:00 p.m., the calves were separated from the cows, during which time they were housed in a pen with access to water. The cows were milked at 5:00 a.m. of the following day immediately after the application of 2 mL of Oxytocin (10 IU/mL, Ocitopec^®^, Biovet, São Paulo, Brazil) in the mammary vein. The milk was weighed after complete extraction and about 30 mL of milk was separated from each cow to assess milk composition. The exact order and time that each cow was milked was recorded and then the calves were kept away from their mothers and a new milking was performed at 6:00 pm to obtain 24 h milk production. After the second milking, the calves were returned to the cows.

The collected milk was analyzed for protein, fat, lactose, and total solids content by infrared spectroscopy (Foss MilkoScan FT120, São Paulo, Brazil).

### 2.6. Blood Samples

For evaluation of the metabolic and hormonal profile, blood samples were collected at 7:00 a.m. on days −21, 0, 21 and 42 from calving (taking parturition as day 0) and for progesterone blood samples were collected at 42 days postpartum by jugular vein puncture, using sterile vacuum tubes and coagulation accelerator gel (SST II Advance^®^, BD Vacuntainer, São Paulo, Brazil) to quantify the concentrations of urea, total proteins, albumin, triglycerides, total cholesterol, non-esterified fatty acids (NEFA), beta-hydroxybutyrate (βHB), and progesterone. A tube with EDTA and sodium fluoride (BD Vacutainer^®^ Fluorinated/EDTA, São Paulo, Brazil) was used to quantify the plasma glucose concentration. After collection, samples were centrifuged at 3600× *g* for 20 min. Serum and plasma were immediately frozen at −20 °C until analyzed. 

Blood samples were analyzed using Bioclin^®^ (Belo Horizonte, Brazil) kits to dose urea (K056-UV kinetic method), total protein (K031-Biuret Method), albumin (K040-Bromocresol Green Method), triglycerides (K117-Enzymatic Colorimetric Method), total cholesterol (K083-Enzymatic Colorimetric Method) and glucose (K082-Enzymatic Colorimetric Method (GOD-PAP)). Non-esterified fatty acids (NEFA) and β-hydroxybutyrate (βHB) were analyzed using Randox^®^ kits (FA115—Colorimetric Method and RB1007—Enzymatic Method, Antrim, UK). An automated biochemical analyzer (Mindray, BS200E, Shenzhen, China) was used for all the aforementioned analyses. Progesterone levels were assessed using a Beckman Coulter^®^ kit (33,550, Brea, CA, USA). Globulins were calculated as the difference between total protein and albumin. Blood urea nitrogen (BUN) was estimated as 46.67% of total serum urea.

### 2.7. Statistical Analysis

The experiment was conducted and analyzed in an entirely randomized experimental design with a double error structure. The results were analyzed by adopting the initial body weight as covariate. The analyses of variance (ANOVA) for the variables studied were performed according to the following mathematical model:

Yijk = μ+T_i + e_(i)j + ε_(ij)k,(1)

where Yijk: observation taken on individual k in paddock j subjected to treatment i; μ: overall mean; Ti: fixed effect of treatment; e(i)j: random, unobservable error associated with each paddock j subjected to treatment i, NID assumption (0, σe^2^), and ε(ij)k: unobservable random error associated with each observation k allocated to paddock j and subjected to treatment i, NID assumption (0, σ^2^ε).

The effect of supplementation and the linear and quadratic effects of supplementation level were evaluated by decomposition of the sum of squares through orthogonal contrasts [16]. Body weight, body composition, and blood metabolite measurements were analyzed using the repeated measures procedure, where the day of collection was considered the repeated variable. The most appropriate covariance structure was chosen based on the lowest value of the corrected Akaike information criterion. For variables that showed a linear or quadratic effect, a Dunnett test at 5% probability was performed to identify whether a supplemented treatment differed from the control. The pregnancy rate was evaluated using a chi-square test. Values of *p* < 0.1 for reproductive evaluations were considered a tendency. For all statistical procedures, the PROC MIXED procedure of SAS (Statistical Analysis System; version 9.4) was used, adopting α = 0.05 as the critical level for the probability of occurrence of type I error.

## 3. Results

The average availability of DM and pdDM (Potentially digestible dry matter) during the experimental period was 4087 and 2970 kg/ha, respectively. For the forage samples collected via simulated manual grazing, the average CP content was 77.7 g/kg DM (Table 2).

**Figure 2:**
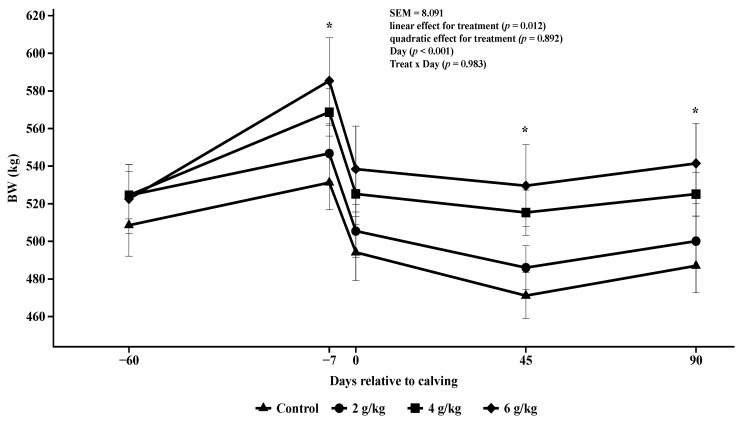
Body weight (BW) during the pre and postpartum period. Asterisks (*) indicate significant differences between treatments (*p* < 0.05).

The BW of the cows showed a positive linear behavior in pre and postpartum with the supplementation plan during the experimental period and showed differences in relation to the day of weighing (*p* = 0.012; *p* < 0.001; Figure 2), and the animals that received 4 and 6 g/kg BW showed differences compared to the animals in the control treatment, in relation to changes in BW 7 days before calving, with greater body weight gain (*p* = 0.008; Table 3), and for these treatments the animals showed less change in body weight at 90 days postpartum (*p* = 0.032; Table 3; Figure 2), compared to the animals of the control treatment. The animals in the 4 and 6 g/kg BW treatments did not differ from each other. Animals in the 2 g/kg BW treatment did not differ from the control treatment for BW change at pre and postpartum (*p* > 0.05; Table 3; Figure 2).

The results for BCS at birth and at 90 days postpartum presented a positive linear effect with increasing supplementation (*p* = 0.015 and *p* = 0.011, respectively; Table 3), and the change in BCS presented a positive linear effect in the prepartum (*p* = 0.007; Table 3), being higher for animals in the treatments 4 and 6 g/kg BW compared to control, however, there was no difference for BCS change 90 days Postpartum (*p* = 0.296; Table 3). For the measurements of LEA (*p* = 0.009), TSR (*p* = 0.001), and TSC (*p* = 0.008) there was a positive linear effect as the supply of supplement increased in the prepartum (Table 3).

Regarding calf performance, no effect of the supplementation plan was observed on birth weight (*p* = 0.744; Table 3) and at 90 days of age (*p* = 0.246; Table 3). There were no differences for overall pregnancy (*p* = 0.9386; Table 4), and for pregnancy at first FTAI, there was a tendency for greater pregnancy as the supplement increased (*p* = 0.07; Table 4), however, a positive linear effect was observed for the service period (*p* = 0.02; Table 3), in which a lower service period was observed for animals in the 4 and 6 g/kg CP treatments compared to animals in the control treatment (*p* < 0.05; Table 3), and animals in the 2 g/kg CP treatment did not differ from the control (*p* > 0.05; Table 3). Milk yield (*p* = 0.698; Table 5) and composition were not influenced by treatments (*p* > 0.05; Table 5).

For the concentrations of BUN (*p* < 0.001; Table 6), βHB (*p* = 0.013; Table 6), and NEFA (*p* = 0.001; Table 6), there was an interaction of treatment with the day of blood collection for the other metabolites there were differences only regarding the day of collection (*p* < 0.01; Table 6).

**Figure 3:**
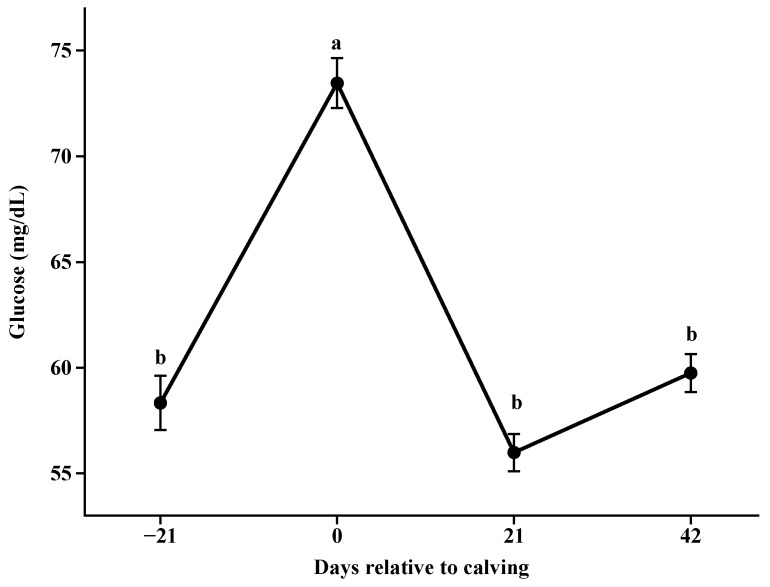
Glucose concentrations during pre and postpartum. Different letters indicate significant differences between collection days (*p* < 0.05).

**Figure 4:**
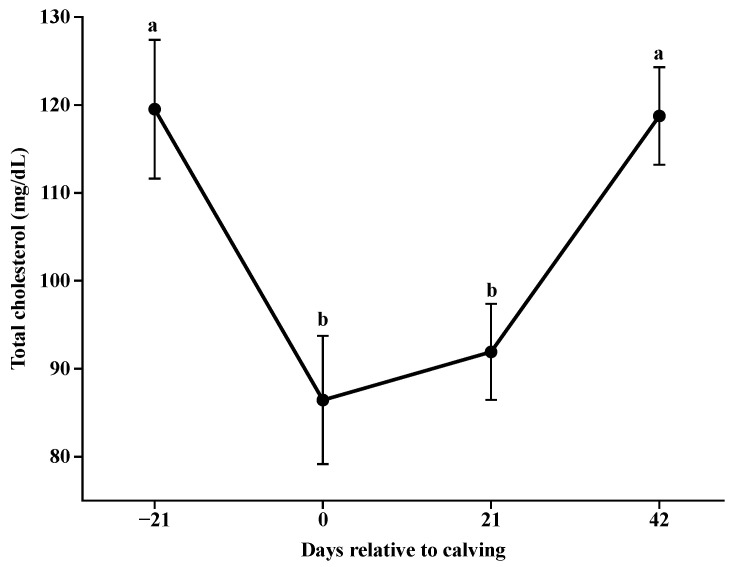
Concentrations of total cholesterol during the pre and postpartum period. Different letters indicate significant differences between collection days (*p* < 0.05).

For glucose (Figure 3), higher concentrations were observed at parturition (*p* < 0.001; 73.5 mg/L), at 21 days postpartum, the concentrations reduced and remained stable (*p* > 0.05). Lower serum concentrations were observed for total cholesterol (Figure 4) at parturition and at 21 days postpartum (*p* < 0.001), which then increased from day 21 to day 42.

**Figure 5:**
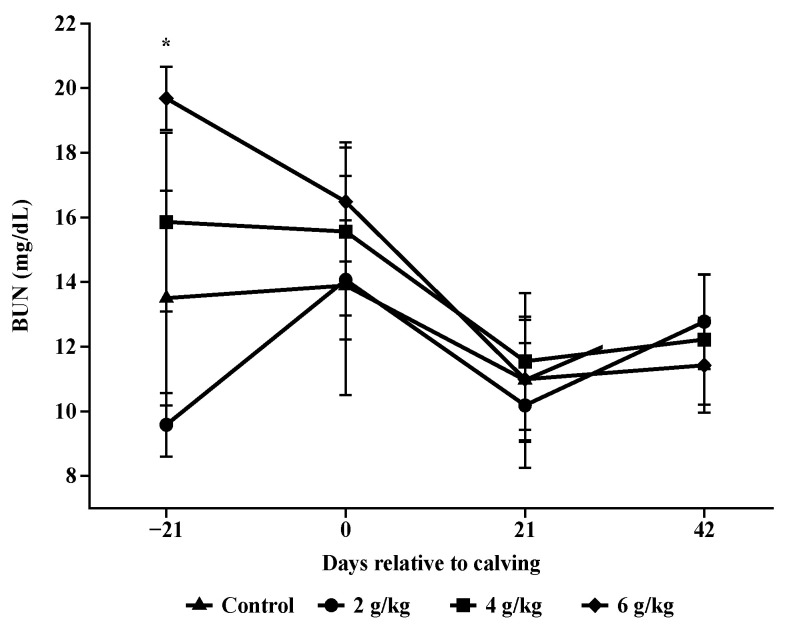
Blood urea nitrogen (BUN) concentrations during pre and postpartum. Asterisks (*) are significantly different between treatments (*p* < 0.05).

The highest concentrations of BUN were observed in cows that received 6 g/kg of PC on day −21, compared to those that received 4 g/kg of PC (*p* = 0.0124; Figure 5), both of which differed significantly from the control group (*p* < 0.001). The animals in the control treatment showed an increase in BUN concentrations at parturition (*p* < 0.001). In contrast, cows in the 6 g/kg PC treatment experienced a reduction in BUN concentrations at parturition (*p* < 0.001). The 2 g/kg and 4 g/kg PC treatments maintained stable BUN concentrations at parturition (*p* = 0.6806 and *p* = 0.7785, respectively).

**Figure 6:**
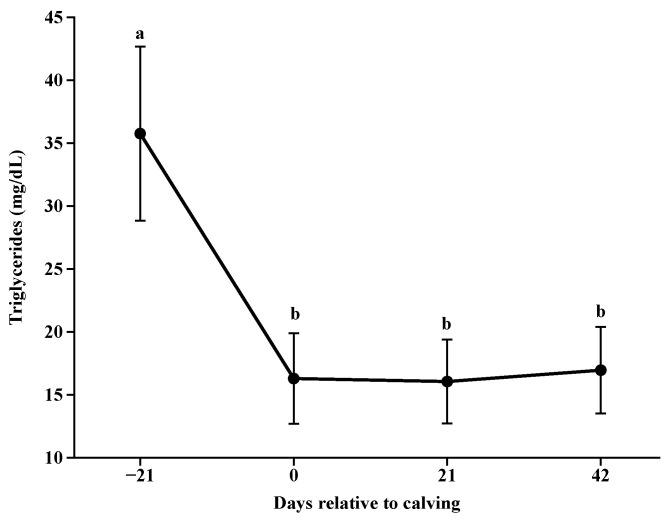
Triglyceride concentrations during pre and postpartum. Different letters indicate significant differences between collection days (*p* < 0.05).

Triglyceride concentrations reduced from day −21 until calving (*p* < 0.0001; Figure 6), holding constant over time (*p* > 0.05; Figure 6).

**Figure 7:**
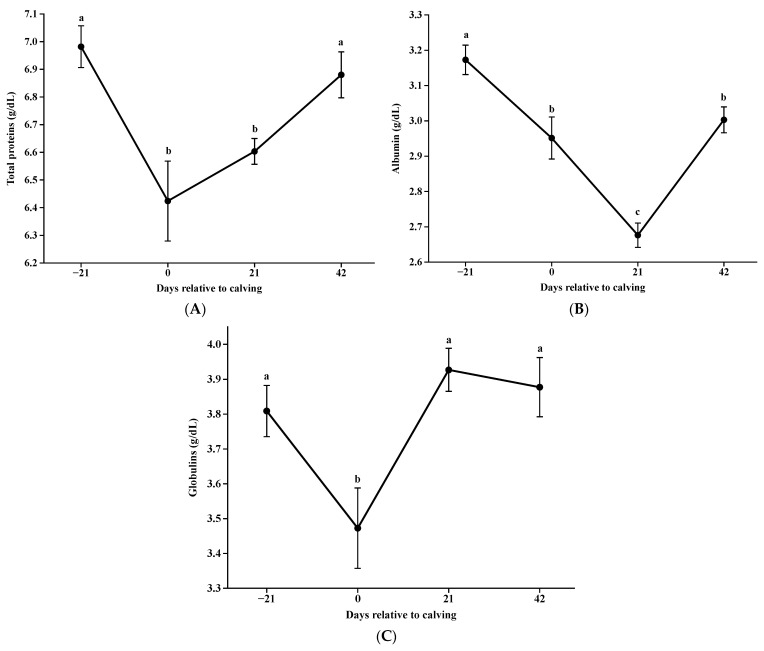
Concentrations of total proteins (**A**); albumin (**B**); globulins (**C**) during pre and postpartum. Different letters indicate significant differences between days (*p* < 0.05).

Total protein concentrations decreased at parturition (*p* < 0.001; Figure 7A), being no difference 21 days postpartum (*p* = 0.2254; Figure 7A) and thereafter increased with the days until day 42 (*p* = 0.0099; Figure 7A). Albumin decreased until day 21 postpartum and subsequently increased (*p* < 0.05; Figure 7B), whereas globulins decreased only at parturition (*p* = 0.004; Figure 7C) and maintained similar concentrations on days −21, 21, and 42 postpartum (*p* > 0.05; Figure 7C).

**Figure 8:**
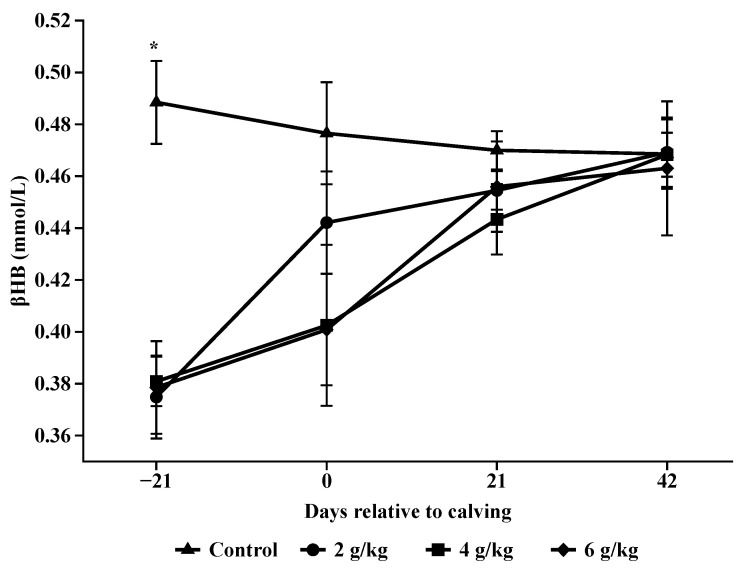
Concentrations of β-hydroxybutyrate (βHB) during pre and postpartum. Asterisks (*) are significantly different between treatments (*p* < 0.05).

The concentration of βHB was higher in the prepartum period for animals in the control treatment (*p* < 0.001; Table 6; Figure 8). In this same period, there was no increase in βHB concentrations for animals that received 4 and 6 g/kg of BW (*p* = 0.5062 and *p* = 0.5142, respectively). However, animals that received 2 g/kg of BW at parturition showed an increase (*p* = 0.0317). After parturition, all supplemented treatments showed an increase in βHB concentrations (*p* < 0.001, Table 6 and Figure 8).

**Figure 9:**
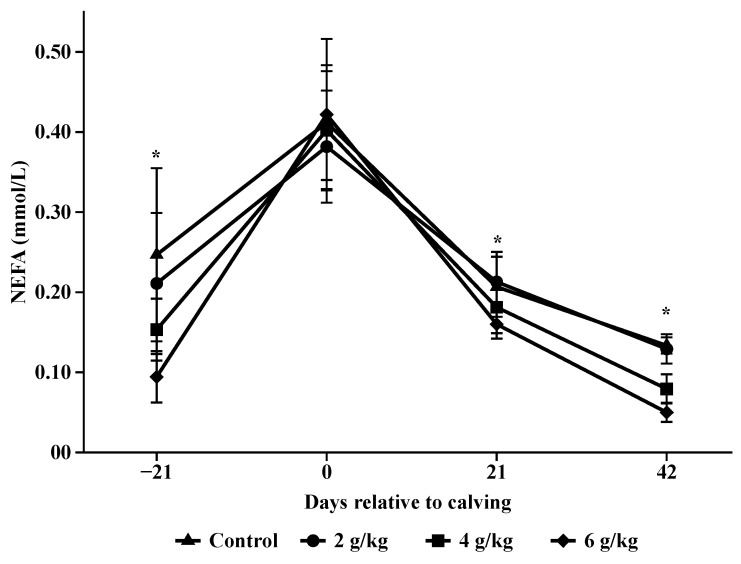
Concentrations of non-esterified fatty acids (NEFA) during pre and postpartum. Asterisks (*) are significantly different between treatments (*p* < 0.05).

The NEFA concentrations were lower 21 days before calving for the animals in the treatments that received 4 and 6 g/kg BW (*p* = 0.004 and *p* = 0.001, respectively) compared to the animals in the control treatment at calving the concentrations were high and there were no differences between the treatments and at 21 days after calving, the 4 and 6 g/kg BW treatments differed from the control (*p* = 0.048 and *p* = 0.031, respectively; Figure 9), and at 42 days after calving the behavior was maintained, with the 4 and 6 g/kg treatments differing from the control (*p* = 0.002 and *p* < 0.001, respectively; Figure 9) the same behavior of reduced NEFA concentrations were observed after calving for all treatments (*p* < 0.001; Figure 9).

**Figure 10:**
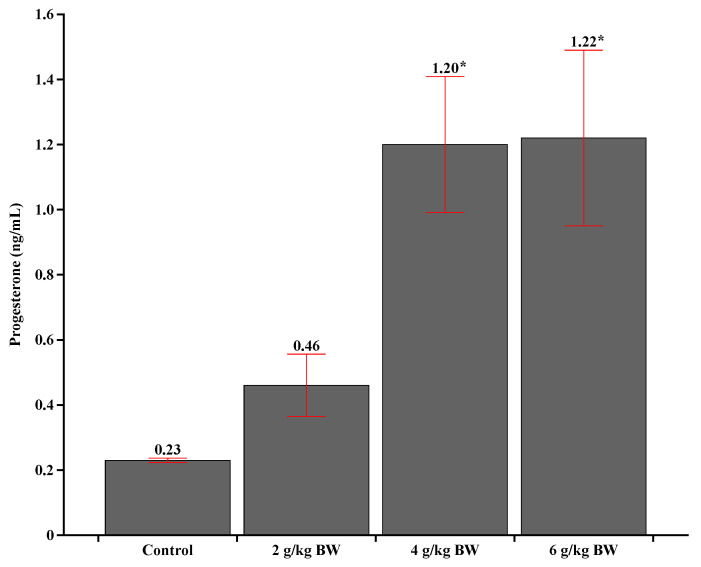
Concentrations of progesterone at 42 days postpartum. Asterisks (*) are significantly different between treatments (*p* < 0.05).

Progesterone concentrations showed a positive linear effect with increasing supplement supply (*p* = 0.009; Table 6; Figure 10), and higher progesterone concentration was observed at 42 days for animals receiving 4 and 6 g/kg BW compared to animals in the treatment 2 g/kg BW (*p* = 0.035 and *p* = 0.034, respectively; Table 6; Figure 10), animals in the 2 g/kg BW treatment did not differ from the control (*p* = 0.394; Table 6 and Figure 10).

## 4. Discussion

In this study the availability of forage was not a limiting factor for animal performance, considering the supply of pdDM of 55 g/kg of animal BW during the entire experimental period, higher than the range suggested by [7] of 40 to 50 g/kg BW. This justifies the good performance of the animals in the control treatment and the animals that received 2 g/kg BW.

During the last 60 days of pregnancy, cows have higher nutritional needs [17] and, according to [18], supplementation administered in adequate amounts during this period can have beneficial effects on the energy and protein metabolism of cows, not only due to the increase in body reserves but also due to its effects on cow metabolism later when supplements are no longer provided. These metabolism results found, provide evidence that adopting higher levels of supplementation in the prepartum period improves postpartum cow performance.

The levels of supplementation in the prepartum period influenced the body weight (BW) at 45 days postpartum. The animals that presented the greatest change in BW during the prepartum period, specifically those that received treatments of 4 and 6 g/kg BW, showed a reduction in BW loss at 90 days postpartum. This suggests a lower negative energy balance (NEB), evidenced by the lower concentration of non-esterified fatty acids (NEFA). Similar results were observed in studies, where animals that received 1.5 kg of supplement (3 g/kg BW) for 60 days before calving also showed less weight loss [18].

For BUN, the concentrations observed for the animals supplemented in the prepartum period were linear with an increase in the level of supplementation. This is due to the fact that BUN is synthesized in the liver in quantities proportional to the concentration of ammonia produced in the rumen and the blood concentration of BUN is directly correlated with the levels of protein, the proportions of rumen degradable protein (RDP) and rumen undegradable protein (RUP), in the supplement and with the energy/protein ratio in the diet [19,20,21]. It is known that feeding high levels of RDP reduces the efficiency of nitrogen recirculation [22,23], leading to a higher concentration of BUN and kidney losses.

Previous studies in cattle during pregnancy [11,24] have provided evidence that supplementation systems during the last third of pregnancy can alter calf birth weight, suggesting that the source of energy and protein may affect fetal growth, which could lead to problems with dystocia. Calf birth weight is a major factor in increasing the risk of dystocia [25] and in such cases, calves typically perform inferiorly compared to animals that have had non-dystocia births [25]. Furthermore, dystocia usually leads to delayed return to estrus by cows postpartum [24].

However, in this study, there were no differences in BW of calves at birth and no cases of dystocia. The performance of calves presented no differences at 90 days post-partum. Corroborating the results of this study, refs. [18,26] found no difference in calf BW at birth according to the supplementation applied to the cows.

According to some studies [4,27], BCS is a determining factor for cows to return to early estrus with better conception rates. Furthermore, for cows with adequate BCS in the final third of pregnancy, there is evidence that body reserves can be utilized during pregnancy without compromising subsequent reproductive function [3].

The literature data reveal that restricted intake in pregnant cows during the late gestation period results in weight loss, body condition score (BCS) loss, and elevated serum concentrations of non-esterified fatty acids (NEFA) and beta-hydroxybutyrate (βHB). This leads to prolonged periods of negative energy balance (NEB) in the postpartum period for both dairy [28,29] and beef cows [27]. Non-esterified fatty acids are mobilized from adipose tissue to provide energy for specific needs [29]. Elevated levels of NEFA can result in the production of ketone bodies, such as β-OHB, and other energy substrates [2].

According to [10], non-supplemented pregnant cows can metabolize energy reserves and alter their metabolism to meet the energy requirements of the growing fetus without altering intake or overall growth, an example of these mechanisms is an increase in circulating βHB accompanied by a reduction in body fat. As observed in this study, βHB concentrations differed between treatments at prepartum with higher concentrations for the non-supplemented animals and lower fat thickness in the rib and croup compared to the other supplemented treatments.

Elevated βHB concentrations are indicative of a lack of energy and nutrients in the animal body, leading to mobilization of its body reserves, resulting from poor adaptation to NEB [30]. In beef cows, reduced serum βHB concentration prior to breeding was associated with increased pregnancy rates at first service [27].

Although there was a difference between treatments in βHB concentrations on day 21, these levels do not indicate a strong mobilization of body reserve for the non-supplemented animals as observed by [2] who found values greater than 0.71 mmol/L of βHB at 30 days prepartum, but rather that the cows had different nutrient balances during prepartum as also observed by [12], in which it was evident for animals in the control and 2 g/kg BW treatment, which also presented greater weight loss postpartum.

The interval between calving and conception greatly influences the profitability of beef production. Thus, in beef cattle systems, it is important that the cow is pregnant within 85 days of calving to ensure that the cow produces one calf per year [18]. Although no effect was observed on the overall pregnancy rate of cows, it is observed that animals that received 4 or 6 g/kg BW of supplement had a higher number of pregnant animals at the first FTAI. In addition, they had a shorter service period (Table 3), which can impact future calf performance, as calves born earlier in the calving season have higher BW at weaning [31].

Prepartum supplementation of beef cows has shown significant positive effects on postpartum progesterone levels. This study observed that higher levels of prepartum supplementation led to higher postpartum progesterone levels and a trend towards a higher pregnancy rate at the first fixed-time artificial insemination (FTAI). Research indicates that energy-protein supplementation during the last 60 to 84 days of pregnancy not only improves productive performance but also optimizes the metabolic status of cows, resulting in higher blood concentrations of progesterone after calving [32,33]. This increase in progesterone levels is directly associated with a faster return to ovarian activity, a crucial factor for fertility and reproductive efficiency in cows. Additionally, adequate supplementation helps to reduce NEFA levels, improving the energy balance and overall health of cows during the peripartum period [32].

The fact that cows that received 4 and 6 g/kg BW, lost less weight from calving to 45 days postpartum in relation to control treatment cows’ evidence that they were less affected with NEB. Furthermore, postpartum NEFA concentrations reduced with time in relation to calving, suggesting recovery of the nutritional status of the animals, also observed by the variation in BW of the animals, but for the animals that received 4 and 6 g/kg BW supplement, these concentrations at postpartum were lower than the control treatment. Thus, these differences in blood concentrations between treatments reinforce that supplementation with 4 and 6 g/kg BW during the prepartum period influenced the energy requirements and the intensity of postpartum catabolism of cows, resulting in higher NEB for non-supplemented animals and animals of the 2 g/kg BW treatment, as previously discussed which having a higher BW. In fact, the animals in the treatments 4 and 6 g/kg BW, obtained higher levels of progesterone at 42 days after calving in relation to the animals in the control treatment, which resulted in lower NEB and consequently lower loss BW of these animals and shorter period of service.

Total cholesterol concentrations were reduced at calving and increased progressively with days for all nutritional plans. Corroborating with [34,35] for postpartum blood cholesterol in lactating beef cows and the reduction in triglyceride concentrations postpartum suggests their use as energy demand for lactation, as they are important sources of long-chain fatty acids for milk fat synthesis [36].

Albumin concentrations decreased significantly at parturition until 21 days after calving, which could be related to the higher requirement of amino acids for milk production [37]. At calving, globulins presented lower levels compared to the rest of the period, which is justified by the transfer of immunity to colostrum production [38], which is also reflected in the behavior of total protein concentrations.

In view of the results found in the present study, all nutritional plans presented an acceptable calving interval. However, the animals that received 4 and 6 g/kg BW supplementation presented a shorter service period compared to the control and 2 g/kg BW groups. Thus, the increase in earlier calving due to earlier pregnancy can increase the value of calves at weaning, justifying a higher supplementation level. In this case, there is no significant difference between the use of 4 and 6 g/kg BW in supplements. Therefore, economically, the use of 4 g/kg BW is the most recommended.

## 5. Conclusions

Providing 4 g/kg BW of protein-energy supplement to grazing Nellore cows 60 days prior to calving is recommended, which improves the metabolic characteristics of NEFA, βHB, and progesterone levels and performance in the prepartum and postpartum period, potentially affecting pregnancy rates.

## Figures and Tables

**Table 1:** Supplement composition (g/kg) as feed.

Item	Supplement
Ingredients (g/kg)	
Ground corn	258
Wheat bran	258
Soybean meal	479
Urea:ammonium sulfate (9:1)	5

Mineral mixture composition: dicalcium phosphate (500.0 g/kg), sodium chloride (471.9 g/kg), zinc sulfate (15.0 g/kg), copper sulfate (7.0 g/kg), cobalt sulfate (0.5 g/kg), potassium iodide (0.5 g/kg), sodium selenite (0.1 g/kg) and manganese sulfate (5.0 g/kg).

**Table 2:** Chemical composition of the supplement and forage.

Item	Supplement	Forage				
		August	September	October	November	December
DM ^1^	892.3	651.2	665.4	516	234.4	238.6
OM ^2^	965	931.8	933.6	934.7	933.7	934.1
CP ^2^	308.9	54.9	51.3	62.5	100.2	102.3
apNDF ^2^	163.3	749.2	780.6	709.4	530.3	535.1
iNDF ^2^	56.7	339.6	352.1	330.8	166.9	170.5

DM—Dry matter, OM—Organic matter, CP—Crude protein, apNDF—neutral detergent fiber corrected for ash and protein, iNDF—indigestible neutral detergent fiber. ^1^ g/kg of natural material. ^2^ g/kg of DM.

**Table 3:** Effect of supplementation levels on cow and calf performance.

Item ^1^	Treatments (g/kg BW)				SEM	*p*-Value ^2^		
	Control	2	4	6		C × S	L	Q
Cows								
Initial BW, kg	508	524	524	522	16.62	0.465	0.599	0.607
BW 7 days prepartum, kg	531	547	569	585	17.04	0.140	0.071	0.974
Calving BW, kg	494	505	525	538	16.79	0.205	0.110	0.956
BW 45 days Postpartum, kg	471	486	515	530	15.21	0.087	0.038	0.983
BW 90 days Postpartum, kg	487	500	525	541	15.82	0.202	0.055	0.920
^3^ Change BW prepartum, kg	22.6	22.3	44.2 *	62.7 *	6.577	0.053	0.008	0.209
BW change 45 days Postpartum, kg	−22.9	−19.6	−9.9	−8.9	5.478	0.183	0.839	0.101
BW change 90 days Postpartum, kg	−7.1	−5.5	0.0 *	2.7 *	2.428	0.091	0.032	0.815
Initial BCS	5.9	6.0	6.1	6.0	0.149	0.401	0.589	0.423
Calving BCS	6.4	6.8	7.5 *	7.7 *	0.164	0.007	0.015	0.454
BCS 90 days Postpartum	5.5	5.8	6.3 *	6.7 *	0.208	0.032	0.011	0.789
Change of prepartum BCS	0.51	0.77	1.4 *	1.65 *	0.179	0.021	0.007	0.977
BCS change 90 days Postpartum	−0.83	−0.97	−1.15	−0.89	0.126	0.296	0.575	0.169
LEA, cm^2^	42.6	45.6	48.5 *	49.8 *	1.171	0.015	0.009	0.479
TSR, mm	2.6	3.2	4.2 *	4.9 *	0.238	0.004	0.001	0.854
TSC, mm	3.2	3.6	4.9 *	5.4 *	0.382	0.028	0.008	0.894
Serving period, days	81	76	57 *	56 *	5.749	0.054	0.020	0.784
Calves								
BW Birth, kg	37	36	35	37	1.754	0.744	0.878	0.396
BW 90 days, kg	87	91	89	95	3.091	0.246	0.155	0.666
ADG, kg/d	0.97	1.01	0.99	1.06	0.347	0.252	0.157	0.653

^1^ BW—Body weight; BCS—Body Condition Score; LEA—Loin Eye Area; TSR—thickness of subcuta-neous rib fat; TSC—thickness of subcutaneous fat on the croup; FTAI—fixed-time artificial insemination; ADG—Average daily gain. ^2^ C × S—Control vs. Supplementation; L e Q—linear and quadratic order effects. * Means statistically different from the control by Dunnett’s test. ^3^ Change in body weight 7 days prepartum compared to the start of the experiment

**Table 4:** Effect of supplementation levels on cow reproduction.

Item ^1^	Treatments (g/kg BW)				SEM	*p*-Value
	Control	2	4	6		
First FTAI pregnancy, %	33.3	45.4	70	88.9	-	0.0701
Overall pregnancy, %	77.8	81.8	88.9	88.9	-	0.9386

^1^ FTAI—fixed-time artificial insemination.

**Table 5:** Effect of supplementation levels on milk yield and composition.

Item	Treatments (g/kg BW)				SEM	*p*-Value ^1^		
	Control	2	4	6		C × S	L	Q
Production, kg/d	7.5	7.8	7.6	7.7	0.439	0.698	0.825	0.831
Fat, %	5.6	5.5	5.3	5.5	0.337	0.691	0.749	0.697
Protein, %	4.5	4.5	4.5	4.4	0.129	0.859	0.711	0.726
Lactose, %	4.5	4.5	4.5	4.6	0.104	0.674	0.548	0.853
Total solids, %	15.2	15.3	15.0	15.2	0.376	0.981	0.924	0.919

^1^ C × S—Control vs. Supplementation; L e Q—linear and quadratic order effects.

**Table 6:** Effect of supplementation levels on the metabolic profile of cows in pre and postpartum.

Item ^1^	Treatments (g/kg BW)				SEM	*p*-Value ^2^				
	Control	2	4	6		C × S	L	Q	Day	Treat × Day
Glucose, mg/dL	61.3	61.9	62.9	61.2	1.338	0.948	0.921	0.416	<0.0001	0.489
BUN, mg/dL	11.6	12.9	13.7 *	14.6 *	0.635	0.060	0.040	0.771	<0.0001	<0.001
Total Cholesterol, mg/dL	100.3	106	108	100.5	3.096	0.363	0.893	0.151	<0.0001	0.995
Triglycerides, mg/dL	20.2	22.1	22.3	20.1	0.924	0.291	0.970	0.084	<0.0001	0.160
Total Protein, g/dL	6.8	6.7	6.7	6.5	0.114	0.354	0.230	0.792	0.0002	0.871
Albumin, g/dL	2.9	2.9	3.0	3.0	0.051	0.282	0.150	0.697	<0.0001	0.707
Globulins, g/dL	3.9	3.8	3.7	3.6	0.127	0.188	0.100	0.956	0.003	0.996
βHB, mmol/L	0.47	0.43 *	0.42 *	0.42 *	0.011	0.015	0.020	0.105	<0.0001	0.013
NEFA, mmol/L	0.25	0.23	0.20	0.18	0.017	0.033	0.010	0.782	<0.0001	0.001
Progesterone, ng/mL	0.22	0.46	1.19 *	1.22 *	0.179	0.023	0.009	0.584	-	-

^1^ BUN—Blood urea nitrogen; βHB—β-hydroxybutyrate; NEFA—Non-esterified fatty acids. ^2^ C × S—Control vs. Supplementation; L e Q—linear and quadratic order effects; Day related to the birth; Treat × Day—Interaction treatment day. * Means statistically different from the control by Dunnett’s test.

## Data Availability

The data presented in this study are available upon request from the corresponding author.

## References

[1] Baruselli P.S., Reis E.L., Marques M.O., Nasser L.F., Bó G.A. (2004). The use of hormonal treatments to improve reproductive performance of anestrous beef cattle in tropical climates. Anim. Reprod. Sci..

[2] Mulliniks J.T., Kemp M.E., Endecott R.L., Cox S.H., Roberts A.J., Waterman R.C., Petersen M.K. (2013). Does β-hydroxybutyrate concentration influence conception date in young postpartum range beef cows. J. Anim. Sci..

[3] Diskin M.G., Kenny D.A. (2016). Managing the reproductive performance of beef cows. Theriogenology.

[4] Hess B.W., Lake S.L., Scholljegerdes E.J. (2005). Nutritional controls of beef cow reproduction. J. Anim. Sci..

[5] Sartori R., Guardieiro M.M. (2010). Nutritional factors associated with reproduction in the female bovine. Rev. Bras. Zootec..

[6] Detmann E., Paulino M.F., de Campos Valadares Filho S., Huhtanen P. (2014). Nutritional aspects applied to grazing cattle in tropics: A review based on Brazilian results. Semin. Ciências Agrárias.

[7] Paulino M.F., Figueiredo D.M., Moraes E.H.B.K. (2004). Supplementation of cattle on pasture: A systemic view. Symposium on Beef Cattle Production.

[8] Ayres H., Ferreira R.M., Torres-Júnior J.R.D.S., Demétrio C.G.B., Sá Filho M.F.D., Gimenes L.U., Baruselli P.S. (2014). Inferences of body energy reserves on conception rate of suckled Zebu beef cows subjected to timed artificial insemination followed by natural mating. Theriogenology.

[9] Santos J.E.P., Cerri R.L.A., Sartori R. (2008). Nutritional management of the donor cow. Theriogenology.

[10] Wood K.M., Awda B.J., Fitzsimmons C., Miller S.P., McBride B.W., Swanson K.C. (2013). Influence of pregnancy in mid-to-late gestation on circulating metabolites, visceral organ mass, and abundance of proteins relating to energy metabolism in mature beef cows. J. Anim. Sci..

[11] Payne J.M., Payne S. (1987). The Metabolic Profile Test.

[12] de Lana Ferreira M.F., Rennó L.N., Detmann E., Paulino M.F., de Campos Valadares Filho S., Moreira S.S., Martins H.C., de Oliveira B.I.C., Marquez J.A., de Paula Cidrine I. (2020). Performance, metabolic and hormonal responses of grazing Nellore cows to an energy-protein supplementation during the pre-partum phase. BMC Vet. Res..

[13] De Almeida D.M., Marcondes M.I., Rennó L.N., Martins L.S., Contreras D., Saldarriaga F.V., Villadiego F.A.C., Ortega R.M., Moreno D.P.S., Moura F.H. (2020). Effects of pre- and postpartum supplementation on lactational and reproductive performance of grazing Nellore beef cows. Anim. Prod. Sci..

[14] National Research Council—NRC (2016). Nutrient Requirements of Beef Cattle.

[15] Detmann E., Costa e Silva L.F., Palma M.N.N. (2021). Methods for Food Analysis—INCT—Ciência Animal.

[16] Steel R.G.D., Torrie J.H., Dicky D.A. (1997). Principles and Procedures of Statistics, A Biometrical Approach.

[17] Valadares Filho S.D.C., Costa e Silva L.F., Gionbelli M.P., Rotta P.P., Marcondes M.I., Chizzotti M.L., Prados L.F. (2016). BR-CORTE 3.0—Nutrient Requirements of Zebu and Crossbred Cattle.

[18] Silva A.G., Paulino M.F., Detmann E., Fernandes H.J., da Silva Amorim L., Ortega R.E.M., Bitencourt J.A. (2017). Energetic-protein supplementation in the last 60 days of gestation improves performance of beef cows grazing tropical pastures. J. Anim. Sci. Biotechnol..

[19] Chalupa W., Sniffen C.J. (1996). Protein and amino acid nutrition of lactating dairy cattle—Today and tomorrow. Anim. Feed. Sci. Technol..

[20] Schroeder G.F., Titgemeyer E.C. (2008). Interaction between protein and energy supply on protein utilization in growing cattle: A review. Livest. Sci..

[21] Wittwer F., Opitz H., Reyes J., Contrras P.C., Bohmwald H. (1993). Diagnóstico de desbalance nutricional mediante la determinación de urea em muestras de leche de rebaños bovinos. Arch. Med. Veterinária.

[22] Marini J.C., van Amburgh M.E. (2003). Nitrogen metabolism and recycling in Holstein heifers. J. Anim. Sci..

[23] Marini J.C., Klein J.D., Sands J.M., van Amburgh M.E. (2004). Effect of nitrogen intake on nitrogen recycling and urea transporter abundance in lambs. J. Anim. Sci..

[24] Patterson D., Bellows R., Burfening P., Carr J. (1987). Occurrence of neonatal and postnatal mortality in range beef cattle. I. Calf loss incidence from birth to weaning, backward and breech presentations and effects of calf loss on subsequent pregnancy rate of dams. Theriogenology.

[25] Boakari Y.L., Ali H.E.-S., Hopper R.M. (2021). Management to Prevent Dystocia. Bovine Reproduction.

[26] Summers A.F., Meyer T.L., Funston R.N. (2015). Impact of supplemental protein source offered to primiparous heifers during gestation on I. Average daily gain, feed intake, calf birth body weight, and rebreeding in pregnant beef heifers. J. Anim. Sci..

[27] Marques R.S., Cooke R.F., Rodrigues M.C., Moriel P., Bohnert D.W. (2016). Impacts of cow body condition score during gestation on weaning performance of the offspring. Livest. Sci..

[28] Barber M.C., Clegg R.A., Travers M.T., Vernon R.G. (1997). Lipid metabolism in the lactating mammary gland. Biochim. Biophys. Acta.

[29] Bauman D.E., Cronje P.B. (2000). Regulation of nutrient partitioning during lactation: Homeostasis and homeorhesis revisited. Ruminant Physiology, Digestion, Metabolism, Growth and Reproduction, Proceedings of the 9th International Symposium on Ruminant Physiology.

[30] Herdt T.H. (2000). Ruminant adaptation to negative, energy balance. Influences on the etiology of ketosis and fatty liver. Vet. Clin. N. Am. Food Anim. Pract..

[31] Funston R.N., Musgrave J.A., Meyer T.L., Larson D.M. (2012). Effect of calving distribution on beef cattle progeny performance. J. Anim. Sci..

[32] Leonhardt S.A., Edwards D.P. (2002). Mechanism of action of progesterone antagonists. Exp. Biol. Med..

[33] Mann G.E., Fray M.D., Lamming G.E. (2006). Effects of time of progesterone supplementation on embryo development and interferon-τ production in the cow. Vet. J..

[34] Godoy M.M.D., Alves J.B., Monteiro A.L.G., Valério Filho W.V. (2004). Reproductive and metabolic parameters of Guzerá cows supplemented in pre and postpartum. Rev. Bras. Zootec..

[35] Reist M., Koller A., Busato A., Kupfer U., Blum J.W. (2000). First ovulation and ketone body status in the early postpartum period of dairy cows. Theriogenology.

[36] Aeberhard K., Bruckmaier R.M., Blum J.W. (2001). Metabolic, enzymatic and endocrine status in high yielding dairy cows—Part 2. J. Vet. Med. Ser..

[37] Contreras P., Fhd G., Barcellos J.O., Ospina H., Lao R. (2000). Indicators of protein metabolism used in herd metabolic profiles. Metabolic Profiling in Ruminants: Its Use in Nutrition and Nutritional Diseases.

[38] Weaver D.M., Tyler J.W., VanMetre D.C., Hostetler D.E., Barrington G.M. (2000). Passive transfer of colostral immunoglobulins in calves. J. Vet. Intern. Med..

